# Consultation patterns of children and adolescents with knee pain in UK general practice: analysis of medical records

**DOI:** 10.1186/s12891-017-1586-1

**Published:** 2017-06-02

**Authors:** Zoe A. Michaleff, Paul Campbell, Joanne Protheroe, Amit Rajani, Kate M. Dunn

**Affiliations:** Arthritis Research UK Primary Care Centre, Research Institute for Primary Care and Health Sciences, Keele University, Keele, Staffordshire, ST5 5BG UK

**Keywords:** Child, Adolescent, Knee pain, Musculoskeletal pain, Epidemiology, Medical record data

## Abstract

**Background:**

Knee problems are common in children and adolescents. Despite this, little is known about the epidemiology of knee problems in children and adolescents who consult in general practice. The aim of this study was to describe consultations by children and adolescents about knee problems in general practice, and examine patterns of patient presentations and consultations by age group, sex and area of socio-economic deprivation.

**Methods:**

Consultations records specific to the knee region were extracted from a general practice consultation database (CiPCA) over a one year period. Knee consultation codes were organised into ‘symptom’ or ‘diagnosis’ (sub-categorised: ‘trauma’, ‘non-trauma’) categories. Descriptive statistics were used to describe patient presentations and number of consultations overall, and stratified analysis carried out on age group, sex, and ar﻿ea of socio-economic deprivation.

**Results:**

Out of all musculoskeletal consultations, knee problems were the fourth most common patient presentation, responsible for the second highest number of consultations. Patient presentations and consultations increased up to age 12–15 years and then stabilised. Symptoms codes e.g. ‘knee pain’ were used more commonly than diagnosis codes e.g. ‘knee sprain’ overall. However, symptom code use declined as age increased, more symptom codes were used in girls compared to boys, and more diagnosis codes were used in patients from areas of high socio-economic deprivation.

**Conclusions:**

This study provides insight into the epidemiology of knee problems in children and adolescents in general practice. Future research is needed to improve our understanding of the knee problems encountered by GPs, and the influence socio-economic deprivation has on consultations.

**Electronic supplementary material:**

The online version of this article (doi:10.1186/s12891-017-1586-1) contains supplementary material, which is available to authorized users.

## Background

Musculoskeletal problems are one of the leading causes of years lived with disability and are associated with significant individual, social and financial burden worldwide [1]. While there is extensive research into musculoskeletal problems in adults, our current understanding of the epidemiology, burden and treatment of musculoskeletal problems in children and adolescents is much more limited [2, 3]. Surveys show that up to 50% of children and adolescents report musculoskeletal pain in any 1 month, and while many of these conditions are assumed to be self-limiting, as many as 50% of this population will experience chronic or recurrent pain [4, 5]. The impact of musculoskeletal pain on children and adolescents can extend beyond their normal daily routine of school, social and sports participation, and result in medication use, health care seeking and substantial health care costs [6–9].

General practitioners (GP) are usually the first to assess, treat and manage children with musculoskeletal problems. Nationally representative data from Australia, Spain and the UK suggest that the annual consultation prevalence of musculoskeletal problems in children and adolescents is between 4 and 8% [7, 10, 11]. These studies consistently identified lower limb problems, in particular foot and knee problems, to be the most common body sites children consulted their GP about, regardless of age group or sex. Interestingly, knee problems were the only body region for which boys were consistently more likely to consult than girls [7, 10, 12]. While this suggests that there is a difference in consultation rates between boys and girls repeat consultations are often discounted in these studies and they do not provide any information as to the actual clinical caseload (i.e. total number of consultations) knee problems in children and adolescents have for GPs and it is not known whether the same sex trends would be identified. Considering the poor long-term prognosis of knee pain in this population it is particularly important to account for repeat consultations and the impact these have on GPs clinical caseload.

There is currently limited evidence and understanding about the general population prevalence of healthcare consultations for knee problems by children and adolescents in general practice and the characteristics of these consultations. This includes uncertainty as to how consultations are recorded by GPs (i.e. use of a specific diagnosis code that can explain a patients presenting problem e.g. ‘patella dislocation’, compared to a non-specific symptom code that provides no information on the diagnosis or cause of the problem e.g. ‘knee pain’), the extent to which knee problems are diagnosed by GPs and, whether recording of consultations vary for patients of differing age, sex or socio-economic deprivation. Such information may be important in term of the implications for patient’s management in primary care and their prognosis. The diagnosis of knee problems is often a challenge for health practitioners for a number of reasons including non-specific symptoms such as ‘knee pain’ being commonly reported by patients and indicative of a wide range knee problems and clinical tests either being of limited diagnostic value or not available (e.g. ‘patellofemoral pain’ is a common condition in adolescents and is based on a ‘diagnosis of exclusion’) [13–15]. The way in which GPs code consultations for example using a symptom code such as ‘knee pain’ or specific diagnosis code such as ‘patella dislocation’ has previously been explored in adults with patellofemoral disorders [16–18]. This study found that symptom codes, rather than specific diagnosis codes, were more commonly used by GPs regardless of age but whether or not this finding also applied to children and adolescents, a population in whom lower limb injury is more common [19], is yet to be determined. An improved understanding of the epidemiology of consultations for knee problems in children and adolescents can inform training and assessment priorities in general practice, identify gaps in service availability [20], explore the extent to which GP’s diagnose knee problems in children and adolescents, identify groups of people who consult more frequently and inform the development of new and novel intervention and preventative strategies. The aim of this study was to describe the epidemiology of consultations for knee problems that children and adolescents aged between 3 and 19 years consult for in general practice. Specifically, this study aims to report on how consultations for knee pain are recorded by GPs for this population, and examine the patterns of Read codes used (symptom or diagnostic Read code) for both *patient presentations* and the *number of consultations*, stratified by age group, sex and area of socio-economic deprivation.

## Methods

### Setting and population

The Consultation in Primary Care Archive (CiPCA), is a high quality, validated medical record database, that contains anonymised consultation data from 14 general practices in North Staffordshire, UK [21, 22]. These practices cover a large geographical area representative of the UK general practice population, and CiPCA has shown analogous trends for musculoskeletal conditions in comparison to other large UK based consultation databases [21] and produced comparable estimates of prevalence for musculoskeletal conditions as international databases [23]. General practitioners are a first contact clinician for health services, with over 97% of the population registered with a GP in the UK [10, 24]. Information obtained during a consultation is recorded in the patient’s medical record using Read codes, a hierarchical coding system that uses a standardised set of clinical terms to allow the recording of patient symptoms, diagnoses, procedures, and morbidity in UK general practice [25]. Consultation data from participating practices is collated to form the CiPCA database. Practices that contribute data to CiPCA undergo an annual cycle of assessment, feedback and training in the use of Read codes in order to improve data quality [22]. Examples include assessment of coding completeness in patient records; feedback of coding completeness at the practice, professional group, and individual levels and general training on the use of Read codes (including detail of code used) and request to enter at least one Read code per contact with the patient. However no training is provided to GPs or practice nurses for the assessment or recording of knee problems specifically.

All musculoskeletal consultations recorded in CiPCA in 2010 (1st January 2010 to 31^st^ December 2010) for children and adolescents aged between 3 and 19 years old were extracted. Patients under 3 years old were excluded as consultations for musculoskeletal problems in general practice are rare in this age group [7, 26]. The upper age range (19 years) was chosen based on the World Health Organisation’s definition of an “adolescent” which is a person aged 10 to 19 years old. Eleven practices involved in CiPCA in 2010 contributed data for this study, with a total age eligible practice population of 27 432. Ethical approval for CiPCA was granted by the North Staffordshire Research Ethics Committee (ref 03/04), with extensions approved by Staffordshire and Black Country Research Ethics Committee.

### Read code identification and categorisation

Musculoskeletal Read codes were included from Chapter N “Musculoskeletal and connective tissue diseases”, R “Symptom, signs and ill-defined conditions”, S “Injury and poisoning”, and 1 “History / symptoms” as outlined in previous methodology [10]. All relevant Read codes were then assigned to one of 48 body regions again following previous methodology [10]. Read codes can be accessed from: www.keele.ac.uk/mrr.

For this study, consultations specific to the knee region were identified and extracted. Two clinicians (ZM, physiotherapist *and* JP, general practitioner) then independently categorised the list of knee Read codes into “symptom” or “diagnosis: trauma and non-trauma” categories (Percentage agreement was 82%, Kappa = 0.682 (95%CI 0.615, 0.749), *P* < .0005 [27]). Any disagreements in categorising Read codes were resolved by discussion and consultation with a third author as required until consensus was reached. “*Symptom*” codes used by GPs are recordings of the experience (e.g. pain, ache, swollen knee) but do not include specific information on diagnosis or cause of a problem. A “*diagnosis”* is a specific label used by a healthcare professional to explain a patient’s symptom or sign (e.g. fractured patella, meniscal tear). The “diagnosis” category was further sub-categorised into “*trauma*” or “*non-trauma*” categories. A diagnosis was categorised as “*trauma*” if the most likely mechanism of injury was the result of an acute physical trauma or injury (e.g. fracture, meniscal tear, patella dislocation) [20]. A “*non-trauma diagnosis*” included any apophysitis (e.g. Osgood-schlatter disease) or repetitive, overuse injury (e.g. tendinopathy) [11, 20, 26].

### Stratification variables

Age at time of consultation was derived from the patient’s date of birth linked to the consultation data; sex was also recorded and linked to this data. Age was grouped into four categories based on the stages of development and ability to report musculoskeletal pain: 3–7 years: pre-pubertal and able to report musculoskeletal pain, 8–11 years: onset of puberty and able to reliably report musculoskeletal pain, 12–15 years: puberty and 16–19 years: late adolescent with increasing health autonomy [28–30]. For area of socio-economic deprivation, the Index of Multiple Deprivation (IMD) score was used. The IMD score combines seven domain indices (income deprivation, employment deprivation, education, skills and training deprivation, health deprivation and disability, crime, barriers to housing and services and living environment deprivation) to produce a national measure of overall relative socio-economic deprivation in the UK [31]. Home address postcodes were used to derive a socio-economic deprivation score for each patient; socio-economic deprivation scores ranged from 1 (area of high socio-economic deprivation) to 32 844 (area of low socio-economic deprivation). The socio-economic deprivation variable was formed into three groups (20% low socio-economic deprivation, 60% middle socio-economic deprivation and 20% high socio-economic deprivation) following and previous methodology [32–35].

### Statistical analysis

Descriptive characteristics of patients, consultation frequency (over 1 year period), mean age, sex and socio-economic deprivation are described for those who consulted for musculoskeletal problems and knee problems. Two approaches were used to describe consultation patterns. Firstly, *patient presentations* report the number of patients who consulted, with each patient only counted once even if they consulted on more than one occasion. Secondly, *number of consultations* report the total number of consultations. This combined approach provides details about the actual number of patients consulting for knee problems, as well as an indication of the clinical caseload for GPs. It also provides information on what groups (age, sex, socio-economic deprivation) are more likely to consult on multiple occasions. Descriptive data are provided for patient presentations and the number of consultations by category of Read code used (symptom codes, diagnosis codes: sub-categorised trauma, non-trauma), stratified by age group, sex and socio-economic deprivation. Exploratory analyses of association were conducted using chi-square to test for differences between the category of Read code used and each stratified group (no analysis was carried out on the sub-category of trauma / non-trauma as cell numbers were considered too low, *n* < 5) [36]. For chi-squared tests that contained more than two groups (i.e. age and area of socio-economic deprivation) adjusted standardised residuals were calculated to indicate which groups deviated from the expected frequency (i.e. adjusted residual >1.96). Based on the results of the chi square tests further exploratory *post-hoc* analyses were performed to assess the independent association of each stratification variable within a multivariable model (i.e. controlling for the effect of each variable on each other). Two multivariable logistic regression models were conducted for 1) *patient presentations* and *2) number of consultations* reporting Odds Ratios (OR) and 95% Confidence Intervals (95% CI). Receiving a diagnosis code was the outcome in both models and the independent variables were age group (reference category: 3–7 years), sex (reference category: girls), area of socio-economic deprivation (reference category: area of low socio-economic deprivation), adjusted for practice variability. All statistical analyses were conducted using IBM SPSS Statistics 21.

## Results

### Patient presentations and the number of consultations for knee problems in the context of musculoskeletal problems

In 2010, a total of 5081 musculoskeletal consultations were recorded for 2836 children and adolescents aged 3 to 19 years; this represents an annual consultation prevalence of 1034 per 10,000 registered persons (95% Confidence interval (CI) 998, 1070). Of these, 550 consultations (10.8%) in 327 patients were for a knee problem (annual consultation prevalence of 119 per 10,000 registered persons; 95% CI 107, 133). Consultations were recorded using 42 of the possible 375 knee Read codes available (11.2%). The most frequently used Read codes to record consultations per symptom and diagnosis category are reported in Table 1 and Read codes per sex, age and socio-economic deprivation group are reported in supplementary Additional file 1: Tables S1-S9. Knee problems were the fourth most common anatomical region patients consulted for (patient presentations: foot 11.7%; head 10.4%; hand 9.8%; knee 9.7% of patients who consulted), but accounted for the second most number of consultations (consultations: hand 11.2%, knee 10.8%, foot 10.7% of all musculoskeletal consultations). Characteristics of patients who consulted at least once for musculoskeletal problems and knee problems in 2010 are presented in Table 2.

**Table 1:** The most frequently used Read codes to record consultations per symptom and diagnosis category

Symptom (*n* = 354)		Diagnosis			
		Trauma (*n* = 175)		Non-trauma (*n* = 21)	
Read term (Read code)	No. of consultations (%)	Read term (Read code)	No. of consultations (%)	Read term (Read code)	No. of consultations (%)
Knee pain (1 M10)	177 (32.2)	Other knee injury (SK170)	97 (17.6)	Synovitis of knee (N220z, N220V)	5 (0.9)
Anterior knee pain (N094W)	66 (12.0)	Knee sprain (S54, S54y)	21 (3.8)	Chondromalacia patellae (N074)	4 (0.7)
Arthralgia of knee (N094M)	59 (10.7)	Patella-recurrent dislocation (N0836, N083p)	19 (3.5)	Infrapatella bursitis (N2166)	2 (0.4)
Knee joint pain (N0946)	32 (5.8)	Dislocation of knee NOS (S46z)	8 (1.5)	Prepatellar bursitis (N2165)	2 (0.4)
Swollen knee (16J4)	8 (1.5)	Open #-sublux knee joint (S4F3)	8 (1.5)	Disorder of patella unspecified (N09AX)	2 (0.4)
Clicking knee (N099C)	4 (0.7)	Fracture patella (S32)	7 (1.3)	Illiotibial band bursitis (N2159)	1 (0.2)
Effusion of knee (N090M)	3 (0.5)	Contusion knee (SE411, SE412)	6 (1.1)	Patellofemoral maltracking (N07Y6)	1 (0.2)
Locked knee (N07Y5)	3 (0.5)	Haemarthrosis of the knee (N0916)	3 (0.5)	Knee arthritis NOS (N0626)	1 (0.2)
Knee gives way (N0966)	1 (0.2)	Acute meniscal tear (S460, S462)	2 (0.4)	Patellar tendinitis (N2164)	1 (0.2)
Other symptoms knee (N096M)	1 (0.2)	Meniscus derangement NEC (N082)	1 (0.2)	Discoid lateral meniscus (N071B)	1 (0.2)
-	-	Superficial injury of knee NOS (SD6y2)	1 (0.2)	Reactive arthropathy of knee (N01wB)	1 (0.2)
-	-	Open wound of knee (SA100)	1 (0.2)	-	-
-	-	Degloving injury knee (SA130)	1 (0.2)	-	-

**Table 2:** Characteristics of *patient presentations* for patients aged 3 to 19 years who consulted for musculoskeletal problems and knee problems in 2010

	Musculoskeletal Read code	Knee Read code
Patients	2836	327
One consultation, *n* (%)	1730 (61.0)	226 (69.1)
Two, *n* (%)	588 (20.7)	52 (15.9)
Three, *n* (%)	245 (8.6)	22 (6.7)
Four, *n* (%)	125 (4.4)	11 (3.4)
Five or more, *n* (%)	148 (5.2)	16 (4.9)
Total No. consultations (mean, range)	5 081 (1.8, 1–16)	550 (1.7, 1–12)
Mean age (SD)	12.4 (4.7)	13.4 (4.1)
3 to 7 years, *n* (%)	553 (19.5)	36 (11.0)
8 to 11 years, *n* (%)	546 (19.3)	49 (15.0)
12 to 15 years, *n* (%)	862 (30.4)	139 (42.5)
16 to 19 years, *n* (%)	875 (30.9)	103 (31.5)
Sex:		
Boy, *n* (%)	1480 (52.2)	193 (59.0)
Girl, *n* (%)	1356 (47.8)	134 (41.0)
Area of Socio-economic deprivation		
Low, *n* (%)	578 (20.4)	80 (24.5)
Medium, *n* (%)	1610 (56.8)	181 (55.4)
High, *n* (%)	516 (18.2)	53 (16.2)
Missing, *n* (%)	132 (4.7)	13 (4.0)

Data provided by eleven CiPCA practices from the 27 432 registered patients aged 3 to 19 years old

### Observable trends in consultation data and when stratified by age group, sex and area of socio-economic deprivation

**Fig. 1:**
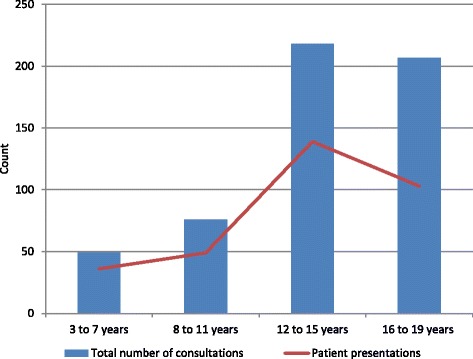
Number of patients and total number of consultations for a knee problem by age group. Patient presentations: Number of patients who consulted about a knee problem on at least one occasion in 2010 i.e. each patient was only counted once even if they consulted on more than one occasion, *n* = 327; Total number of consultations: Total number of consultations for a knee problem in 2010 i.e. includes every clinical contact that was coded with a knee related Read code, *n* = 550

Patient presentations and consultations for knee problems increased with age, with an increase between the 8 and 11-year age group and 12 to 15-year age group, after which there was a small decline in the 16 to 19-year age group, see Fig. 1. Overall, knee-related symptoms codes were used more commonly than diagnosis codes to record both patient presentations and consultations across all age and sex groups; the only exception to this was for patients from areas of high socio-economic deprivation in which diagnosis codes were more frequently used, see Tables 3 and 4. Various trends however were found when examining the proportion of symptom and diagnosis codes used to record consultations for the various age, sex, and area of socio-economic deprivation groups. For example, for both patient presentation and total number of consultations the proportion of symptom codes declined, and the proportion of diagnosis codes used increased, with increasing age, see Tables 3 and 4. For all stratifications (age group, sex, area of socio-economic deprivation) trauma codes were used more commonly than non-trauma codes (29.7% vs. 3.7% overall).

**Table 3:** Category of Read codes used to record knee problems for *patient presentations* when stratified by age group, sex and socio-economic deprivation, *n* (%)

		Symptom	Diagnosis		Row total, *n* (%)
			*Trauma*	*Non-trauma*	
Age*	3 to 7 years, *n* (%)	28 (77.8)	8 (22.2)		36 (11.0)
			8 (22.2)	0 (0)	
	8 to 11 years, *n* (%)	35 (71.4)	14 (28.6)		49 (15.0)
			12 (24.5)	2 (4.1)	
	12 to 15 years, *n* (%)	91 (65.5)	48 (34.5)		139 (42.5)
			42 (30.2)	6 (4.3)	
	16 to 19 years, *n* (%)	64 (62.1)	39 (37.9)		103 (31.5)
			35 (34.0)	4 (3.9)	
	Column total, *n* (%)	218 (66.7)	109 (33.3)		327 (100)
			97 (29.7)	12 (3.7)	
Sex^†^					
	Boy, *n* (%)	125 (64.8)	68 (35.2)		193 (59.0)
			61 (31.6)	7 (3.6)	
	Girl, *n* (%)	93 (69.4)	41 (30.6)		134 (41.0)
			36 (26.9)	5 (3.7)	
	Column total, *n* (%)	218 (66.7)	109 (33.3)		327 (100)
			97 (29.7)	12 (3.7)	
Area of socio-economic deprivation^‡^	Low, *n* (%)	59 (73.8)	21 (26.3)		80 (25.5)
			18 (22.5)	3 (3.8)	
	Medium, *n* (%)	125 (69.1)	56 (30.9)		181 (57.6)
			50 (27.6)	6 (3.3)	
	High, *n* (%)	25 (47.2)	**28 (52.8)**		53 (16.9)
			25 (47.2)	3 (5.7)	
	Missing, *n* (%)	9 (69.2)	4 (30.8)		13 (4.0)
			4 (30.8)	0	
	Column total, *n* (%)	209 (66.6)	105 (33.4)		314 (100)
			93 (29.6)	12 (3.8)	

Patient presentations: Number of patients who consulted on at least one occasion in 2010, *n* = 327

Bolded numbers = cells with significant adjusted standardized residuals greater than +1.96 (observed frequency is more than the expected frequency)

NB: Chi Square tests were applied to test for differences between the stratified groups by symptom or diagnosis (no analysis was carried out on the sub-category of trauma / non-trauma as cell numbers were considered too low, *n* < 5). Data for diagnosis: non-trauma are presented for reader interest

* *X* ^2^ = 3.541, df = 3, *p* = 0.315

† *X* ^2^ = 0.765, df = 1, *p* = 0.382

‡ *X* ^2^ = 11.32, df = 2, *p* = 0.003

**Table 4:** Category of Read codes used to record *the total number of consultations* for knee problems when stratified by age group, sex and socio-economic deprivation, *n* (%)

		Symptom	Diagnosis		Row total, *n* (%)
			*Trauma*	*Non-trauma*	
Age*	3 to 7 years, *n* (%)	**39 (79.6)**	10 (20.4)		49 (8.9)
			8 (16.3)	2 (4.1)	
	8 to 11 years, *n* (%)	**57 (75.0)**	19 (25.0)		76 (13.8)
			17 (22.4)	2 (2.6)	
	12 to 15 years, *n* (%)	**152 (69.7)**	66 (30.3)		218 (39.6)
			58 (26.6)	8 (3.7)	
	16 to 19 years, *n* (%)	106 (51.2)	**101 (48.8)**		207 (37.6)
			92 (44.4)	9 (4.3)	
	Column total, *n* (%)	354 (64.4)	196 (35.6)		550 (100)
			175 (31.8)	21 (3.8)	
Sex†					
	Boy, *n* (%)	191 (59.9)	128 (40.1)		319 (58)
			119 (37.3)	9 (2.8)	
	Girl, *n* (%)	163 (70.6)	68 (29.4)		231 (42)
			56 (24.2)	12 (5.2)	
	Column total, *n* (%)	354 (64.4)	196 (35.6)		550 (100)
			175 (31.8)	21 (3.8)	
Area of socio-economic deprivation ‡	Low, *n* (%)	**106 (77.9)**	30 (22.1)		136 (24.7)
			26 (19.1)	4 (2.9)	
	Medium, *n* (%)	180 (65.0)	97 (35.0)		277 (50.4)
			87 (31.4)	10 (3.6)	
	High, *n* (%)	49 (44.1)	**62 (55.9)**		111 (20.2)
			55 (49.5)	7 (6.3)	
	Missing, *n* (%)	19 (73.1)	7 (26.9)		26 (4.7)
			7 (26.9)	0 (0.0)	
	Column total, *n* (%)	335 (63.9)	215 (39.9)		550 (100)
			168 (32.1)	21 (4.0)	

Total number of consultations for knee problem in 2010, *n* = 550

Bolded numbers = cells with significant adjusted standardized residuals greater than +1.96 (observed frequency is more than the expected frequency)

NB: Chi Square tests were applied to test for differences between the stratified groups by symptom or diagnosis (no analysis was carried out on the sub-category of trauma / non-trauma as cell numbers were considered too low, *n* < 5). Data for diagnosis: non-trauma are presented for reader interest

* *X* ^2^ = 27.05, df = 3, *p* < 0.001

† *X* ^2^ = 6.67, df = 1, *p* = 0.01

‡ *X* ^2^ = 30.56, df = 2, *p* < 0.001

### Associations between Read code categories and age group, sex and area of socio-economic deprivation

#### Age group

For *patient presentation*, no significant difference was found between the symptom and diagnosis category used by GPs and age groups (*X* ^2^ = 3.54, df = 3, *p* = 0.315), see Table 3. For *number of consultations*, a significant difference was found between the symptom and diagnosis category used by GPs and age groups (*X* ^2^ = 27.05, df = 3, *p* < 0.001), see Table 4. Inspection of the adjusted standardised residuals indicated the greatest deviation from expectancy for the 16 to 19 year old group compared to all other age groups. Table 4 shows diagnosis codes were used more frequently than symptom codes to record consultations for 16 to 19 year olds when compared to the three younger age groups (3–7 years, 8–11 years, 12–15 years). Symptom codes were used more frequently than diagnosis codes for each of the three younger age groups compared to 16–19 year olds, and this did not differ between the three younger age groups.

#### Sex

For *patient presentation*, no significant difference was found between the symptom and diagnosis category used by GPs and sex (*X* ^2^ = 0.765, df = 1, *p* = 0.382), see Table 3. For *number of consultations*, a significant difference was found between the symptom and diagnosis category used by GPs and sex (*X* ^2^ = 6.67, df = 1, *p* = 0.001), with symptom codes used more frequently for girls, and diagnosis codes used more frequently for boys, see Table 4.

#### Area of socio-economic deprivation

Chi square testing indicated a significant difference between the symptom and diagnosis category used by GPs and area of socio-economic deprivation for both *patient presentations* (*X* ^2^ = 11.32, df = 2, *p* = 0.003) and the *number of consultations* (*X* ^2^ = 30.56, df = 2, *p* < 0.01), see Tables 3 and 4. For *patient presentations*, the adjusted standardised residuals indicated the greatest deviance from expectancy for patients from areas of high area of socio-economic deprivation compared to those from mid and low area of socio-economic deprivation. Residual scores were not significant for those from mid and low area of socio-economic deprivation. For *number of consultations*, the adjusted standardised residuals again indicated high socio-economic deprivation had the greatest deviation from expectancy compared to mid and low area of socio-economic deprivation. Symptom codes were used significantly more frequently for those from areas of low socio-economic deprivation. No significant difference was found for GPs use of symptom or diagnosis Read codes for those from mid areas of socio-economic deprivation.

### Post-hoc multivariable analysis

For *patient presentation*, compared to low area of socio-economic deprivation (reference category) area of high socio-economic deprivation was significantly associated with receiving a diagnosis category of Read code indicating a 2.7 times increase in odds (OR 2.75; 95% CI 1.13 to 6.68), no association was found for mid area of socio-economic deprivation. Within the model, age, sex and practice variation were not associated with the diagnosis category of Read code. Overall the logistic regression model for patient presentation was statistically significant, *X* ^2^ (16, *n* = 327) = 27.68, *p* = 0.035. The model explained 11.7% (Nagelkerke R^2^) of the variance in GPs use of diagnosis codes and correctly classified 69.4% of cases. Results for the *number of consultations* model show three significant effects. For age (where 3–7 is the reference category) being in the 16–19 age category was significantly associated with receiving a diagnosis code (OR 3.54; 95% CI 1.57 to 7.99), however no associations were found for the other age categories. For deprivation (with low socioeconomic as the reference category), those from high (OR 3.75; 95% CI 1.84 to 7.66) and mid area of socio-economic deprivation (OR 1.85; 95% CI 1.10 to 3.11) were more likely to receive a diagnosis Read code. The analysis of sex (girls as the reference category) showed that boys were at an increase in odds of receiving a diagnosis code (OR 1.62; 95% CI 1.08 to 2.43). The logistic regression model for the number of consultations was statistically significant, *X* ^2^ (16, *n* = 550) = 86.88, *p* < 0.001. The model explained 20.9% (Nagelkerke R^2^) of the variance in GPs use of diagnosis codes and correctly classified 72.9% of cases.

## Discussion

This study describes the epidemiology of consultations by children and adolescents who present to general practice with a knee problem. The patterns of Read codes used are reported for both patient presentations and the number of consultations, stratified by age group, sex and socio-economic deprivation. In the context of all musculoskeletal consultations by children and adolescents, the findings indicate that knee problems are the fourth most common reason for a patient to consult, and involve the second highest number of consultations, accounting for approximately 10% of the childhood musculoskeletal caseload for GPs. The results of this study show that GPs infrequently use specific diagnostic Read codes to record consultations for knee problems; however this practice differs dependent on demographic factors such as the patient’s age, sex, and by area of socio-economic deprivation. This may highlight differences in GPs diagnosis patterns and reflect GPs diagnostic uncertainty when recording consultations (e.g. a higher proportion of symptom codes used to record consultations for younger children compared to older children) or indicate that there are differences in the knee problems that children and adolescents consult for based on these socio-demographic factors and further research is needed to explain these findings.

This study provides insight into how child and adolescent patient presentations and consultations for knee problems are recorded by GPs in the UK, and how this varies by demographic and socio-economic factors. Strengths of this study include the use of routinely recorded data from eleven general practices including every patient aged between 3 and 19 years who consulted their GP about a knee problem in 2010. Patient presentations and consultations in this study mirror the reported onset of knee problems by age in other studies (e.g. the reported high point prevalence in adolescents between 12 and 17 years of age has been reported elsewhere [8, 37]). Compared to previous work that describes consultations for lower limb problems by children and adolescent in primary care [7, 26], this current study reports on knee problems specifically and the range of problems children and adolescents consult their GP about, as well as describes the consultation patterns for knee problems in terms age, sex and socio-economic deprivation. In addition this information informs on overall patient presentations for knee problems and describes GP’s overall clinical load. The sample used in this current study is large and representative of the child and adolescent general practice population in the UK, given that over 95% of the UK population are registered with a general practice. Furthermore CiPCA has demonstrated comparability to other national UK general practice databases [21] as well as international databases.

These findings do need to be interpreted in the context of several limitations. While Read codes provide GPs with a structured way in which to record patient consultations, interpretation is limited for two reasons. Firstly, the way in which GPs assess and diagnose patients and record consultations is not standardised (e.g. through the consistent use of a diagnostic classification system), and is likely to lead to variations in how problems are recorded by different GPs [21, 38]. Therefore, it is not currently known if the patterns identified reflect differences in patient presentations or the coding practices of GPs. Secondly, Read codes do not provide any information as to the mechanism of injury or aetiology of the presenting problem or characteristics of the pain presentation (e.g. pain severity, duration, number of pain sites). In terms of socio-economic deprivation, an IMD score was calculated for each individual patient based on their home postcode. While the IMD score is the most useful and commonly used small area measure of deprivation, actual household deprivation is not known (e.g. family income) and within every area there will be individuals who are more deprived and individuals who are not [31]. Lastly, only a small number of consultations were recorded using ‘diagnosis: non-trauma’ codes thereby limiting the identification of trends and conclusions that could be made. The infrequent use of ‘non-trauma’ codes could be a result of the categorisation of Read codes by authors or the way that these codes are used by GPs. The authors were found to have ‘good’ agreement in categorising Read codes, with discrepancies noted for only a few Read codes which have multiple variations for the same code e.g. ‘recurrent dislocation’ and ‘derangement of the knee’. Of the 375 knee codes 105 were categorised by the two authors as non-trauma codes. This suggests that while there are a large number of non-trauma codes available either these conditions are not commonly seen by GPs, the criteria for their use is unclear or that these codes are only used when there is diagnostic certainty and until which time symptom codes may be used.

In many countries including the UK, GPs are first contact practitioners, responsible for diagnosis, treatment, management, and referral of patients for further investigations and healthcare services where necessary. This study identified that symptom codes were most commonly used by GPs to record consultations for knee problems in children and adolescents. Possible reasons for the frequent use of symptom codes include factors related to GPs and their ability to assess and diagnose musculoskeletal conditions, diagnostic uncertainty, knowledge and availability of treatments, time pressures, their own beliefs about paediatric pain, the value and selection of Read codes and continuity between practitioners, and potential stigma associated with a diagnostic ‘label’ [39]. Lastly, many GPs may actively be taking a ‘wait and see’ approach to see if symptoms resolve or a more clear diagnosis evolves through repeat consultations. The ‘coding culture’ has previously been explored in a qualitative study of anxiety and similar work could be conducted in musculoskeletal conditions to better understand how GPs code these conditions and in patients of various ages [39]. The use of diagnosis codes were found to increase with age, and were more frequently used for boys. These findings are likely to reflect true differences in the problems patient’s consult for as previous studies that have identified both increasing age and male sex to be associated with increased injury rates due to a variety of mechanisms including sport, traffic accident, and collision with or being struck by another object [40–42]. The increased use of diagnosis codes in these groups may also reflect improved communication abilities with age and may indicate increased injury severity especially for boys e.g. increased incidence of fractures. These finding combined suggest that the risk factors for age and sex vary and that these need to be considered when developing and targeting prevention strategies. The significance of the type of Read code used to record consultations for knee pain is not yet known and further work could examine whether the type of code (i.e. symptom or diagnosis code) used by GPs has any association with a patients prognosis or the way in which their condition is managed (e.g. treatments and referrals for further investigations or to other health professionals) [43].

A third of patients who consulted for a knee problem in this study (see Table 1) were found to consult on more than one occasion during the study year. For those who consulted more than once, the same Read code category (symptom, diagnosis) was used to record subsequent consultations in 90.1% of cases. This finding suggests that a substantial proportion of patients have a recurrent or persistent knee problem. Previous studies have shown that knee pain persists in a significant proportion of patients (from 33 to 90%) [44–47]. Persistent knee pain in children and adolescents has been found to be associated with high pain intensity, low quality of life and an increased risk of ceasing all participation in sports [8, 46]. The implications of knee pain experienced during childhood and adolescents and long term health conditions (e.g. osteoarthritis) are still to be determined [48], although more recent evidence suggests an association between adolescent knee pain and patellofemoral arthritis [49]. Considering the generally poor prognosis of knee pain experienced during adolescents [46], further work is needed to identify clinically meaningful and modifiable prognostic factors that can enable the early identification of those who are at most risk of recurrent or persistent symptoms.

An interesting finding of this study was the differences in the Read code category used by GP’s to record consultations for patients from areas of high and low socio-economic deprivation, with symptoms codes (e.g. ‘knee pain’) used predominantly for children and adolescents from areas of low socio-economic deprivation and diagnosis codes (e.g. ‘knee sprain’, ‘patella dislocation’) for those from areas of high socio-economic deprivation. The relationship between socio-economic deprivation and the onset, persistence and outcomes for musculoskeletal health in children and adolescents is complex, often conflicting and currently not well understood. This is likely due to variations in study methodology, study and healthcare setting (e.g. country specific and access to healthcare), how socio-economic status defined and is measured (e.g. family level vs. area level; domains incorporated and weightings of each), the types and severity of injuries evaluated and how these are reported (e.g. all vs. stratification of injury by type). For example, a study conducted in Spanish primary care identified an association between low socio-economic status and wounds, bruises, sprains and fractures in boys and girls aged less than 15 years [50]. Similarly, a Scottish study utilising hospital fracture data found both fracture incidence and type of fracture was associated with social deprivation, with high socio-economic deprivation associated with a higher incidence of fracture and fractures occurring in the upper limb [51]. However, data from the Alberta healthcare registry found that while overall injury rates were higher in children from high socio-economic deprivation, the rate of dislocations / sprains and strains was found to vary with socio-economic status (e.g. compared to children who receive no healthcare subsidies the rate of dislocations / sprains and strains was lower in children receiving partial or total health care subsidies however higher for children receiving welfare) and no association was found for fracture [52]. Additional hypothesis driven studies are needed to clarify the relationship between socio-economic deprivation and the types, location and severity of musculoskeletal problems in children and adolescent as well as the influence socio-economic deprivation has overtime in terms of recovery, recurrence and persistence of pain. Further quantitative and qualitative work is also needed to identify the mechanisms and risk factors associated with musculoskeletal problems and how these may differ, or not, along the social gradient. For example, socio-economic deprivation appears to be associated with injuries sustained around the home and during recreation and play but *not* with sports-related injuries [53]. This future work would enable the identification of potentially ‘at risk populations’ and inform the development of tailored and targeted prevention strategies.

## Conclusion﻿s

Knee problems in children and adolescents represent a significant caseload for GPs and the propensity for these problems to persist presents a significant challenge for general practice. This study described the epidemiology of GP consultations for knee problems in children and adolescents aged between 3 and 19 years and in doing so identified several avenues for future research that in time could provide GPs with an evidence-based approach to the management of these problems.

## Additional file

Additional file 1: Table S1. The most frequently used Read codes to record consultations per symptom and diagnosis category for boys (*n* = 319). **Table S2.** The most frequently used Read codes to record consultations per symptom and diagnosis category for girls (*n* = 231). **Table S3.** The most frequently used Read codes to record consultations per symptom and diagnosis category for age group 3 to 7 years (*n* = 49). **Table S4.** The most frequently used Read codes to record consultations per symptom and diagnosis category for age group 8 to 11 years (*n* = 76). **Table S5.** The most frequently used Read codes to record consultations per symptom and diagnosis category for age group 12 to 15 years (*n* = 218). **Table S6.** The most frequently used Read codes to record consultations per symptom and diagnosis category for age group 16 to 19 years (*n* = 207). **Table S7.** The most frequently used Read codes to record consultations per symptom and diagnosis category for areas of high socioeconomic deprivation (*n* = 111). **Table S8.** The most frequently used Read codes to record consultations per symptom and diagnosis category for areas of mid socioeconomic deprivation (*n* = 277). **Table S9.** The most frequently used Read codes to record consultations per symptom and diagnosis category for areas of low socioeconomic deprivation (*n* = 136). (DOCX 36 kb)

## Abbreviations

CiPCA: The Consultation in Primary Care Archive
GP: General practitioner
