# Supplementary material for: Consultation patterns of children and adolescents with knee pain in UK general practice: analysis of medical records
Source: BMC Musculoskelet Disord. 2017 Jun 2;18:239. doi: 10.1186/s12891-017-1586-1 (PMC5457541; doi:10.1186/s12891-017-1586-1)
Supplement: Additional file 1: Table S1. — The most frequently used Read codes to record consultations per symptom and diagnosis category for boys (n = 319). Table S2. The most frequently used Read codes to record consultations per symptom and diagnosis category for girls (n = 231). Table S3. The most frequently used Read codes to record consultations per symptom and diagnosis category for age group 3 to 7 years (n = 49). Table S4. The most frequently used Read codes to record consultations per symptom and diagnosis category for age group 8 to 11 years (n = 76). Table S5. The most frequently used Read codes to record consultations per symptom and diagnosis category for age group 12 to 15 years (n = 218). Table S6. The most frequently used Read codes to record consultations per symptom and diagnosis category for age group 16 to 19 years (n = 207). Table S7. The most frequently used Read codes to record consultations per symptom and diagnosis category for areas of high socioeconomic deprivation (n = 111). Table S8. The most frequently used Read codes to record consultations per symptom and diagnosis category for areas of mid socioeconomic deprivation (n = 277). Table S9. The most frequently used Read codes to record consultations per symptom and diagnosis category for areas of low socioeconomic deprivation (n = 136). (DOCX 36 kb) [file 12891_2017_1586_MOESM1_ESM.docx]

**Supplementary tables:**

**Table 1: The most frequently used Read codes to record consultations per symptom and diagnosis category for boys (n=319)**

| **Symptom** | | **Diagnosis** | | | |
| --- | --- | --- | --- | --- | --- |
|  |  | **Trauma** | | **Non-trauma** | |
| **Read term**  **(Read code)** | **No. of consultations (%)** | **Read term**  **(Read code)** | **No. of consultations (%)** | **Read term**  **(Read code)** | **No. of consultations (%)** |
| Knee pain (1M10) | 91 (28.5) | Other knee injury (SK170) | 70 (21.9) | Prepatella bursitis (N2165) | 2 (0.6) |
| Arthralgia of knee (N094M) | 31 (9.7) | Knee sprain  (S54, S54y) | 15 (4.7) | Illiotibial band bursitis (N2159) | 1 (0.3) |
| Anterior knee pain (N094W) | 30 (9.4) | Open #-sublux knee joint (S4F3) | 8 (2.5) | Infrapatella bursitis (N2166) | 1 (0.3) |
| Knee joint pain (N0946) | 27 (8.5) | Patella-recurrent dislocation (N0836, N083p) | 7 (2.2) | Chondromalacia patellae (N074) | 1 (0.3) |
| Swollen knee (1614) | 8 (2.5) | Fracture patella  (S32) | 6 (1.9) | Discoid lateral meniscus (N071B) | 1 (0.3) |
| Clicking knee (N099C) | 2 (0.6) | Contusion knee (SE411, SE412) | 4 (1.3) | Knee arthritis NOS (N0626) | 1 (0.3) |
| Effusion of knee (N090M) | 2 (0.6) | Haemarthrosis of the knee (N0916) | 3 (0.9) | Patellofemoral maltracking (N07Y6) | 1 (0.3) |
| - | - | Dislocation of knee NOS (S46z) | 2 (0.6) | Reactive arthropathy of the knee | 1 (0.3) |
| - | - | Acute meniscal tear (S460, S462) | 2 (0.6) | - | - |
| - | - | Meniscus derangement NEC (N082) | 1 (0.3) | - | - |
| - | - | Open wound of knee- | 1 (0.3) | - | - |

**Table 2: The most frequently used Read codes to record consultations per symptom and diagnosis category for girls (n=231)**

| **Symptom** | | **Diagnosis** | | | |
| --- | --- | --- | --- | --- | --- |
|  |  | **Trauma** | | **Non-trauma** | |
| **Read term**  **(Read code)** | **No. of consultations (%)** | **Read term**  **(Read code)** | **No. of consultations (%)** | **Read term**  **(Read code)** | **No. of consultations (%)** |
| Knee pain (1M10) | 86 (37.2) | Other knee injury (SK170) | 27 (11.7) | Synovitis of knee (N220z, N220V) | 5 (2.2) |
| Anterior knee pain (N094W) | 36 (15.6) | Patella-recurrent dislocation (N0836, N083p) | 12 (5.2) | Chondromalacia patellae (N074) | 3 (1.3) |
| Arthralgia of knee (N094M) | 28 (12.1) | Knee sprain  (S54, S54y) | 6 (2.6) | Disorder of the patella unspecified | 2 (0.9) |
| Knee joint pain (N0946) | 5 (2.2) | Dislocation of knee NOS (S46z) | 6 (2.6) | Infrapatella bursitis (N2166) | 1 (0.4) |
| Locked knee (N07Y5) | 3 (1.3) | Contusion knee (SE411, SE412) | 2 (0.9) | Patellar tendinitis (N2164) | 1 (0.4) |
| Clicking knee (N099C) | 2 (0.9) | Fracture patella  (S32) | 1 (0.4) | - | - |
| Effusion of knee | 1 (0.4) | Superficial injury of knee NOS | 1 (0.4) | - | - |
| Knee gives way (N0966) | 1 (0.4) | Degloving injury knee | 1 (0.4) | - | - |
| Other symptoms knee | 1 (0.4) | - | - | - | - |

**Table 3: The most frequently used Read codes to record consultations per symptom and diagnosis category for age group 3 to 7 years (n=49)**

| **Symptom** | | **Diagnosis** | | | |
| --- | --- | --- | --- | --- | --- |
|  |  | **Trauma** | | **Non-trauma** | |
| **Read term**  **(Read code)** | **No. of consultations (%)** | **Read term**  **(Read code)** | **No. of consultations (%)** | **Read term**  **(Read code)** | **No. of consultations (%)** |
| Knee pain (1M10) | 20 (40.8) | Other knee injury (SK170) | 8 (16.3) | Synovitis of knee (N220z, N220V) | 1 (2.0) |
| Knee joint pain (N0946) | 6 (12.4) | - | - | Discoid lateral meniscus (N071B) | 1 (2.0) |
| Arthralgia of knee (N094M) | 5 (10.2) | - | - | - | - |
| Anterior knee pain (N094W) | 4 (8.2) | - | - | - | - |
| Swollen knee (1614) | 3 (6.2) | - | - | - | - |
| Clicking knee (N099C) | 1 (2.0) | - | - | - | - |

**Table 4: The most frequently used Read codes to record consultations per symptom and diagnosis category for age group 8 to 11 years (n=76)**

| **Symptom** | | **Diagnosis** | | | |
| --- | --- | --- | --- | --- | --- |
|  |  | **Trauma** | | **Non-trauma** | |
| **Read term**  **(Read code)** | **No. of consultations (%)** | **Read term**  **(Read code)** | **No. of consultations (%)** | **Read term**  **(Read code)** | **No. of consultations (%)** |
| Knee pain (1M10) | 27 (35.5) | Other knee injury (SK170) | 9 (11.8) | Chondromalacia patellae (N074) | 1 (1.3) |
| Arthralgia of knee (N094M) | 14 (18.4) | Knee sprain  (S54, S54y) | 6 (7.9) | Reactive arthropathy of the knee | 1 (1.3) |
| Anterior knee pain (N094W) | 9 (11.8) | Contusion knee (SE411, SE412) | 1 (1.3) | - | - |
| Knee joint pain (N0946) | 6 (7.9) | Dislocation of knee NOS (S46z) | 1 (1.3) | - | - |
| Clicking knee (N099C) | 1 (1.3) | - | - | - | - |

**Table 5: The most frequently used Read codes to record consultations per symptom and diagnosis category for age group 12 to 15 years (n=218)**

| **Symptom** | | **Diagnosis** | | | |
| --- | --- | --- | --- | --- | --- |
|  |  | **Trauma** | | **Non-trauma** | |
| **Read term**  **(Read code)** | **No. of consultations (%)** | **Read term**  **(Read code)** | **No. of consultations (%)** | **Read term**  **(Read code)** | **No. of consultations (%)** |
| Knee pain (1M10) | 90 (41.3) | Other knee injury (SK170) | 35 (16.1) | Infrapatella bursitis (N2166) | 2 (0.9) |
| Anterior knee pain (N094W) | 33 (15.3) | Knee sprain  (S54, S54y) | 7 (3.2) | Disorder of the patella unspecified | 2 (0.9) |
| Arthralgia of knee (N094M) | 18 (8.3) | Patella-recurrent dislocation (N0836, N083p) | 7 (3.2) | Chondromalacia patellae (N074) | 1 (0.5) |
| Knee joint pain (N0946) | 6 (2.8) | Dislocation of knee NOS (S46z) | 4 (1.8) | Illiotibial band bursitis (N2159) | 1 (0.5) |
| Clicking knee (N099C) | 2 (0.9) | Contusion knee (SE411, SE412) | 2 (0.9) | Patellofemoral maltracking (N07Y6) | 1 (0.5) |
| Knee gives way (N0966) | 1 (0.5) | Fracture patella  (S32) | 1 (0.5) | Prepatellar bursitis | 1 (0.5) |
| Effusion of knee | 1 (0.5) | Superficial injury of knee NOS | 1 (0.5) | - | - |
| Other symptoms knee | 1 (0.5) | Open wound of knee | 1 (0.5) | - | - |

**Table 6: The most frequently used Read codes to record consultations per symptom and diagnosis category for age group 16 to 19 years (n=207)**

| **Symptom** | | **Diagnosis** | | | |
| --- | --- | --- | --- | --- | --- |
|  |  | **Trauma** | | **Non-trauma** | |
| **Read term**  **(Read code)** | **No. of consultations (%)** | **Read term**  **(Read code)** | **No. of consultations (%)** | **Read term**  **(Read code)** | **No. of consultations (%)** |
| Knee pain (1M10) | 40 (19.3) | Other knee injury (SK170) | 45 (21.7) | Synovitis of knee (N220z, N220V) | 4 (1.9) |
| Arthralgia of knee (N094M) | 22 (10.6) | Patella-recurrent dislocation (N0836, N083p) | 11 (5.3) | Chondromalacia patellae (N074) | 2 (1.0) |
| Anterior knee pain (N094W) | 20 (9.7) | Knee sprain  (S54, S54y) | 8 (3.9) | Prepatella bursitis (N2165) | 1 (0.5) |
| Knee joint pain (N0946) | 14 (6.8) | Open #-sublux knee joint (S4F3) | 8 (3.9) | Patellar tendinitis (N2164) | 1 (0.5) |
| Swollen knee (1614) | 5 (2.4) | Fracture patella  (S32) | 6 (2.9) | Knee arthritis NOS (N0626) | 1 (0.5) |
| Locked knee (N07Y5) | 3 (1.5) | Dislocation of knee NOS (S46z) | 4 (1.9) | - | - |
| Effusion of knee (N090M) | 2 (1.0) | Contusion knee (SE411, SE412) | 3 (1.5) | - | - |
|  |  | Haemarthrosis of the knee (N0916) | 3 (1.5) | - | - |
| - | - | Acute meniscal tear (S460, S462) | 2 (1.0) | - | - |
| - | - | Meniscus derangement NEC (N082) | 1 (0.5) | - | - |
| - | - | Degloving injury knee | 1 (0.5) | - | - |

**Table 7: The most frequently used Read codes to record consultations per symptom and diagnosis category for areas of high socioeconomic deprivation (n=111)**

| **Symptom** | | **Diagnosis** | | | |
| --- | --- | --- | --- | --- | --- |
|  |  | **Trauma** | | **Non-trauma** | |
| **Read term**  **(Read code)** | **No. of consultations (%)** | **Read term**  **(Read code)** | **No. of consultations (%)** | **Read term**  **(Read code)** | **No. of consultations (%)** |
| Knee pain (1M10) | 29 (26.1) | Other knee injury (SK170) | 23 (20.7) | Disorder of the patella unspecified | 2 (1.8) |
| Anterior knee pain (N094W) | 6 (5.4) | Patella-recurrent dislocation (N0836, N083p) | 14 (12.6) | Chondromalacia patellae (N074) | 2 (1.8) |
| Swollen knee (1614) | 5 (4.5) | Knee sprain  (S54, S54y) | 8 (7.2) | Patellofemoral maltracking (N07Y6) | 1 (0.3) |
| Arthralgia of knee (N094M) | 4 (3.6) | Haemarthrosis of the knee (N0916) | 3 (2.7) | Knee arthritis NOS (N0626) | 1 (0.3) |
| Knee joint pain (N0946) | 3 (2.7) | Dislocation of knee NOS (S46z) | 2 (1.8) | Illiotibial band bursitis (N2159) | 1 (0.3) |
| Other symptoms knee | 1 (0.9) | Acute meniscal tear (S460, S462) | 2 (1.8) | - | - |
| - | - | Fracture patella  (S32) | 1 (0.9) | - | - |
| - | - | Meniscus derangement NEC (N082) | 1 (0.9) | - | - |
| - | - | Open wound of knee- | 1 (0.9) | - | - |

**Table 8: The most frequently used Read codes to record consultations per symptom and diagnosis category for areas of mid socioeconomic deprivation (n=277)**

| **Symptom** | | **Diagnosis** | | | |
| --- | --- | --- | --- | --- | --- |
|  |  | **Trauma** | | **Non-trauma** | |
| **Read term**  **(Read code)** | **No. of consultations (%)** | **Read term**  **(Read code)** | **No. of consultations (%)** | **Read term**  **(Read code)** | **No. of consultations (%)** |
| Knee pain (1M10) | 91 (32.9) | Other knee injury (SK170) | 50 (18.1) | Synovitis of knee (N220z, N220V) | 3 (1.1) |
| Anterior knee pain (N094W) | 35 (12.6) | Knee sprain  (S54, S54y) | 9 (3.2) | Prepatella bursitis (N2165) | 2 (0.7) |
| Arthralgia of knee (N094M) | 33 (11.9) | Open #-sublux knee joint (S4F3) | 8 (2.9) | Chondromalacia patellae (N074) | 2 (0.7) |
| Knee joint pain (N0946) | 13 (4.7) | Fracture patella  (S32) | 6 (2.2) | Reactive arthropathy of the knee | 1 (0.4) |
| Clicking knee (N099C) | 4 (1.4) | Dislocation of knee NOS (S46z) | 6 (2.2) | Infrapatella bursitis (N2166) | 1 (0.4) |
| Locked knee (N07Y5) | 2 (0.7) | Patella-recurrent dislocation (N0836, N083p) | 5 (1.8) | Discoid lateral meniscus (N071B) | 1 (0.4) |
| Knee gives way (N0966) | 1 (0.4) | Superficial injury of knee NOS | 1 (0.4) | - | - |
| Effusion of knee (N090M) | 1 (0.4) | Degloving injury knee | 1 (0.4) | - | - |
| - | - | Contusion knee (SE411, SE412) | 1 (0.4) | - | - |

**Table 9: The most frequently used Read codes to record consultations per symptom and diagnosis category for areas of low socioeconomic deprivation (n=136)**

| **Symptom** | | **Diagnosis** | | | |
| --- | --- | --- | --- | --- | --- |
|  |  | **Trauma** | | **Non-trauma** | |
| **Read term**  **(Read code)** | **No. of consultations (%)** | **Read term**  **(Read code)** | **No. of consultations (%)** | **Read term**  **(Read code)** | **No. of consultations (%)** |
| Knee pain (1M10) | 52 (38.2) | Other knee injury (SK170) | 21 (15.4) | Synovitis of knee (N220z, N220V) | 2 (1.5) |
| Anterior knee pain (N094W) | 24 (17.6) | Contusion knee (SE411, SE412) | 4 (2.9) | Patellar tendinitis (N2164) | 1 (0.7) |
| Arthralgia of knee (N094M) | 21 (15.4) | Knee sprain  (S54, S54y) | 1 (0.7) | Infrapatella bursitis (N2166) | 1 (0.7) |
| Knee joint pain (N0946) | 7 (5.1) | - | - | - | - |
| Locked knee (N07Y5) | 1 (0.7) | - | - | - | - |
| Effusion of knee (N090M) | 1 (0.7) | - | - | - | - |
